# Supplementary material for: EnzML: multi-label prediction of enzyme classes using InterPro signatures
Source: BMC Bioinformatics. 2012 Apr 25;13:61. doi: 10.1186/1471-2105-13-61 (PMC3483700; doi:10.1186/1471-2105-13-61)
Supplement: Addtional file 5 — The Java code to format the data files, evaluate and predict. The file enzml_java_code.tar.gz contains the Java code used to format database data to ARFF and XML formats, to execute cross and train-test (jackknife) evaluations and to record evaluation results to database. More information is included in the readme.txt file and the Javadoc files. The code can be used with a MySQL database. To use a different database software, other JDBC drivers might be required. [file 1471-2105-13-61-S5.gz › java_code/utils/doc/uk/ac/ed/inf/utils/database/TableWriter.html]

TableWriter


---


|  |  |  |
| --- | --- | --- |
| **Overview** \| **Package** \| **Class** \| **Use** \| **Tree** \| **Deprecated** \| **Index** \| **Help** | |  |
| **PREV CLASS**   NEXT CLASS | **FRAMES**    **NO FRAMES**     **All Classes** |  |
| SUMMARY: NESTED \| FIELD \| CONSTR \| METHOD | DETAIL: FIELD \| CONSTR \| METHOD |  |


---


## uk.ac.ed.inf.utils.database Class TableWriter

```
java.lang.Object
  uk.ac.ed.inf.utils.database.DbManaged
      uk.ac.ed.inf.utils.database.TableManaged
          uk.ac.ed.inf.utils.database.TableWriter
```

---

``` public class TableWriter extends TableManaged ```

A TableWriter object contains all the information to create, fill and delete
a database table.

**Version:**
:   24-Mar-2007
    TODO create hierarchy?: table > table from file

**Author:**
:   Luna De Ferrari

---

**Constructor Summary**

`TableWriter(TableManager tableManager)`
  
          Constructor 2: takes the table name and a list of fields


| **Method Summary** | |
| --- | --- |
| `java.lang.String` | `getInsertSql(TableRow row)`             Returns the sql "insert into" statement for the row |
| `java.lang.String` | `getLoadDataInfileSql(java.lang.String fileName, java.lang.String fieldTerminator, java.lang.String lineTerminator, int numberOfInitialLinesToIgnore, java.lang.String commaSeparatedListOfColumns)` |
| `java.lang.Integer` | `insertReturnsKey(TableRow row)`             Executes an sql 'insert into' operation. |
| `boolean` | `writeFileToTable(java.lang.String fileName, java.lang.String fieldTerminator, java.lang.String lineTerminator, int numberOfInitialLinesToIgnore, java.lang.String commaSeparatedListOfColumns)`             Writes a data file to table using LOAD DATA LOCAL INFILE return true if the statement was executed |
| `java.lang.Integer` | `writeRowToTable(TableRow row)`             Insert the row values into the appropriate database table |

**Methods inherited from class uk.ac.ed.inf.utils.database.TableManaged**

`getTableManager`

**Methods inherited from class uk.ac.ed.inf.utils.database.DbManaged**

`getDbConnection, getDbManager`

**Methods inherited from class java.lang.Object**

`equals, getClass, hashCode, notify, notifyAll, toString, wait, wait, wait`

**Constructor Detail**

### TableWriter

```
public TableWriter(TableManager tableManager)
```

:   Constructor 2: takes the table name and a list of fields

    **Parameters:**: `tableManager` - the table manager


**Method Detail**

### getInsertSql

```
public java.lang.String getInsertSql(TableRow row)
```

:   Returns the sql "insert into" statement for the row

    :   **Parameters:**: `row` - **Returns:**: the sql "insert into" statement for the row

---


### getLoadDataInfileSql

```
public java.lang.String getLoadDataInfileSql(java.lang.String fileName,
                                             java.lang.String fieldTerminator,
                                             java.lang.String lineTerminator,
                                             int numberOfInitialLinesToIgnore,
                                             java.lang.String commaSeparatedListOfColumns)
```

---


### insertReturnsKey

```
public java.lang.Integer insertReturnsKey(TableRow row)
```

:   Executes an sql 'insert into' operation. And returns the auto-generated
    key for the inserted row.

    :   **Parameters:**: `row` - a table row (columns + values) **Returns:**: the auto-generated key for the inserted row.

---


### writeFileToTable

```
public boolean writeFileToTable(java.lang.String fileName,
                                java.lang.String fieldTerminator,
                                java.lang.String lineTerminator,
                                int numberOfInitialLinesToIgnore,
                                java.lang.String commaSeparatedListOfColumns)
```

:   Writes a data file to table using LOAD DATA LOCAL INFILE return true if
    the statement was executed

---


### writeRowToTable

```
public java.lang.Integer writeRowToTable(TableRow row)
```

:   Insert the row values into the appropriate database table

    :   **Returns:**: the table auto-generated key for the inserted row (or null if the row was not inserted)


---


|  |  |  |
| --- | --- | --- |
| **Overview** \| **Package** \| **Class** \| **Use** \| **Tree** \| **Deprecated** \| **Index** \| **Help** | |  |
| **PREV CLASS**   NEXT CLASS | **FRAMES**    **NO FRAMES**     **All Classes** |  |
| SUMMARY: NESTED \| FIELD \| CONSTR \| METHOD | DETAIL: FIELD \| CONSTR \| METHOD |  |


---
